# Aging-Related Ovarian Failure and Infertility: Melatonin to the Rescue

**DOI:** 10.3390/antiox12030695

**Published:** 2023-03-11

**Authors:** Russel J. Reiter, Ramaswamy Sharma, Alejandro Romero, Walter Manucha, Dun-Xian Tan, Debora Aparecida Pires de Campos Zuccari, Luiz Gustavo de Almeida Chuffa

**Affiliations:** 1 Department of Cell Systems and Anatomy, Joe R and Teresa Lozano Long School of Medicine, UT Health San Antonio, San Antonio, TX 78229, USA; 2 Department of Pharmacology and Toxicology, Faculty of Veterinary Medicine, Complutense University of Madrid, 28040 Madrid, Spain; 3 Instituto de Medicina y Biologia Experimental de Cuyo (IMBECU), Consejo Nacional de Investigaciones Cientificas y Tecnologicas (CONICET), Mendoza 5500, Argentina; 4 Laboratorio de Investigacao Molecular do Cancer, Faculdade de Medicina de Sao Jose do Rio Preto, Sao Jose do Rio Preto 15080-000, Brazil; 5 Department of Structural and Functional Biology, Institute of Biosciences of Botucatu, Botucatu 18618-689, Brazil

**Keywords:** oxidative stress, oocyte, granulosa cells, sirtuins, inflammation, extrapineal melatonin, reproductive life span, mitochondrial physiology, in vitro fertilization–embryo transfer

## Abstract

Aging has a major detrimental effect on the optimal function of the ovary with changes in this organ preceding the age-related deterioration in other tissues, with the middle-aged shutdown leading to infertility. Reduced fertility and consequent inability to conceive by women in present-day societies who choose to have children later in life leads to increased frustration. Melatonin is known to have anti-aging properties related to its antioxidant and anti-inflammatory actions. Its higher follicular fluid levels relative to blood concentrations and its likely synthesis in the oocyte, granulosa, and luteal cells suggest that it is optimally positioned to interfere with age-associated deterioration of the ovary. Additionally, the end of the female reproductive span coincides with a significant reduction in endogenous melatonin levels. Thus, the aims are to review the literature indicating melatonin production in mitochondria of oocytes, granulosa cells, and luteal cells, identify the multiple processes underlying changes in the ovary, especially late in the cessation of the reproductive life span, summarize the physiological and molecular actions of melatonin in the maintenance of normal ovaries and in the aging ovaries, and integrate the acquired information into an explanation for considering melatonin in the treatment of age-related infertility. Use of supplemental melatonin may help preserve fertility later in life and alleviate frustration in women delaying childbearing age, reduce the necessity of in vitro fertilization–embryo transfer (IVF-ET) procedures, and help solve the progressively increasing problem of non-aging-related infertility in women throughout their reproductive life span. While additional research is needed to fully understand the effects of melatonin supplementation on potentially enhancing fertility, studies published to date suggest it may be a promising option for those struggling with infertility.

## 1. Introduction

Reproductive infertility in both females and males is becoming progressively more common throughout the world [1]. Infertility is a growing public health concern that is acute since most epidemiological studies report a 50% increase in this problem in the last 60 years [2]. It is estimated that 35% of cases in which a couple is unable to conceive are a consequence of ovarian failure with an equal percentage being a result of male infertility. The causes of the failure to produce/shed healthy oocytes are numerous, e.g., hormonal imbalance, poor nutrition, excessive physical or psychological stress, genetic background, ovarian disease, anti-cancer therapies, respiratory pollutants, chemical or heavy metal exposure, advancing age, etc. [3,4,5]. Some of these fall into the category of “endocrine disruptors” (EDCs) [6] and many are the result of industrialization and the introduction of products that contain reproductive toxins which are subsequently inhaled or ingested [7,8]. In addition to significantly reducing fertilization of healthy oocytes and successful pregnancy, there are complex psychological and social consequences associated with failure of conception [9]. In many cases, the basis of female infertility is molecular damage of subcellular molecules at the level of the ovary and related organs that result from the excessive production of reactive oxygen species (ROS); the resulting damage is typically characterized as oxidative stress which functionally interferes with the normal physiology of the oocyte or other mammalian cells.

The loss of the ability to reproduce obviously has serious implications when it occurs prematurely; however, in females, it is also a natural consequence of aging when infertility develops during middle age, well before human females are considered old. Attempts to delay the functional involution of the female reproductive system has attracted significant interest in recent years since in current societies many human females delay having a child to late in their reproductive life, a time when conception may be difficult and the chances of having a child with a specific pathology are increased. One factor related to the shutdown of the reproductive system seems to be the gradual accumulation of molecular damage which depletes the ovary of viable oocytes. Given that melatonin is a ubiquitous and universal antioxidant and the fact that its production has dropped significantly by the time the reproductive system collapses, herein we consider the possibility that the loss of this critical antioxidant may be a contributor to the onset of infertility and that the shutdown may be a means to protect against the development of preeclampsia, miscarriages, fetal pathologies, and other issues.

## 2. Partially Reduced Oxygen Species; Requirements for Normal Ovarian/Oocyte Physiology

Usually, when the concept of ROS (a term often used synonymously with the phrase free radicals) is discussed, the molecular destructive properties of these agents are enumerated [10,11,12]. ROS, which are often formed as by-products of aerobic metabolism when electrons are leaked from the electron transport chain, however, are not always pariahs but are rather sometimes essential signaling molecules for the mediation of critical subcellular events [13,14].

Relative to ovarian and reproductive physiology, ROS have significant positive functional roles in regulating follicular growth and oocyte maturation, ovulation, fertilization of the oocyte by a sperm, successful uterine implantation of the developing embryo and the growth of the fetus throughout pregnancy [15,16,17,18,19]. ROS that are generated by primary, secondary and Graafian follicles aid in follicular growth, maturation of the oocyte and the deterioration of the follicular wall allowing for follicle rupture and shedding of the oocyte [20,21]. For these processes to occur optimally, a delicate balance exists between the degree of free radical production and their incapacitation by radical scavengers or due to their enzymatic degradation [22,23]. Both locally synthesized and pineal-derived melatonin, which arrives via the circulation, presumably contribute to maintaining oxidative homeostasis in the peripheral reproductive system. That melatonin is avidly taken up from the blood by the ovary was documented within a decade of the discovery of this indole [24]; these data in fact suggest that the ovary may be a preferred site for melatonin uptake. It remains a mystery how most beneficial ROS escape from being neutralized by efficient radical scavengers, while those that inflict oxidative stress are simultaneously rendered inactive by the same reducing agents [25]. Whatever the processes involved, for successful reproduction to occur, there must be a finely regulated balance between free radical generation and their removal at all levels of the peripheral reproductive continuum.

Beyond the local factors that interfere with all the essential events between successful gametogenesis and the ultimate delivery of healthy offspring, there are alterations that occur in the hypothalamo-pituitary axis that render animals living under natural environmental conditions incapable of sexually reproducing for several months each year [23,26,27,28]. This neuroendocrine regulation of seasonal sexual quiescence/activity is most obviously manifested in photosensitive seasonally breeding mammals, most of which inhabit the temporal and polar regions of the earth [29,30,31]. These circannual reproductive variations are driven by pineal-derived melatonin signaling [32] rather than occur as a result of any melatonin that may be produced in the neurons of the medio-basal hypothalamus [33,34]; this distant regulation of peripheral reproductive capability is unrelated to the potent antioxidative processes of this multitasking molecule. Although there are reported minor changes in the cell physiology of the gonads and adnexa of humans, the hypothalamic events described here for the regulation of seasonal reproductive capability are not applicable to humans given that the season of mating is generally not considered a factor for successful reproduction in the human [35].

## 3. Melatonin: Redox Mechanisms for Preserving Reproductive Health

Melatonin, a highly functionally diverse, low-molecular-weight antioxidant and anti-inflammatory agent has been sparingly studied relative to its ability to reduce patho-physiological processes common to the peripheral female reproductive system [36,37,38,39,40]. Of special note is that the concentration of melatonin in the follicular fluid of Graafian follicles from healthy females exceeds that in the blood [41,42]; moreover, the levels of melatonin in human follicular fluid reportedly may depend on the season in which it is measured [43], an observation that is in need of confirmation. Oocytes, granulosa cells and luteal cells synthesize melatonin which likely contributes to its concentration in the follicular fluid [44,45,46,47]. In addition, the classic membrane receptors for melatonin, MT1 and MT2, are found in several areas of the ovary, e.g., oocyte, granulosa cells, luteal cells, etc. [48,49,50,51].

In addition to its known receptor-mediated functions, melatonin directly protects all components of the ovary from oxidative damage that is a consequence of locally generated ROS and reactive nitrogen species (RNS) [52,53,54]. As noted in the previous section, ROS in particular sometimes function as essential signaling agents in ovarian reproductive processes including follicle maturation, follicle involution due to normal atresia, ovulation, formation of the corpus luteum, and maturation of both the pre- and post-ovulated oocyte [55]. Excessive ROS generation which accompanies many toxin exposures and aging, however, mutilates critical molecules that compromise the health of the gamete (oocyte) which results in offspring with genetic disorders or in reproductive failure.

Melatonin of pineal origin or locally produced is a potent direct free radical scavenger [56,57,58]; the oxidation potential of melatonin is on the order of +570 mV [59]. The metabolites that are formed when melatonin donates an electron also have the capacity to neutralize partially reduced oxygen species [22,60]. Melatonin also chelates transition metals that participate in generation of the highly toxic hydroxyl radical [61,62], and synergistically acts in concert with other classic radical scavengers to reduce oxidative stress [63,64].

**Figure 1:**
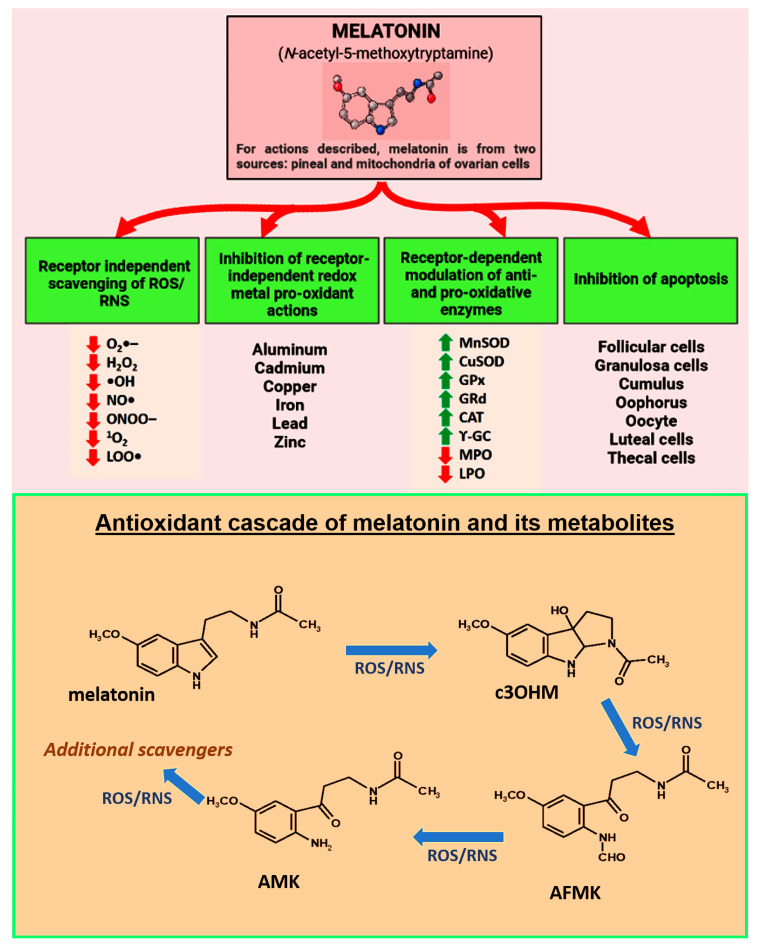
Melatonin, which is nocturnally produced in and secreted by the pineal gland and in a non-circadian manner by the mitochondria of other cells, including those that are components of the ovary, is proposed as a critical factor in protecting against premature infertility and reproductive cessation. Moreover, published evidence indicates that supplementation with melatonin delays ovarian aging in animals and lowers the frequency of infertility in humans. In reference to melatonin’s protective actions against reproductive collapse, the figure summarizes the multiple receptor-independent and receptor-dependent processes that interfere with especially oxidative stress-mediated ovarian deterioration with the critical cells undergoing apoptosis. In addition to directly scavenging ROS/RNS and indirectly lowering oxidative damage by upregulating antioxidative enzymes and downregulating pro-oxidant enzymes, melatonin binds redox reactive metal ions to limit the Fenton and Haber–Weiss reactions thereby reducing the production of the highly toxic hydroxyl radical. Red arrows indicate inhibition; green arrows indicate stimulation. The bottom panel illustrates what has come to be known as melatonin’s antioxidant cascade as a radical scavenger. Thus, not only is melatonin a direct radical scavenger, but so are its metabolites, cyclic 3-hydroxymelatonin (c3OHM), N-acetyl-N-formyl-5-methoxykynuramine (AFMK), N-acetyl-5-methoxykynuramine (AMK) and possibly others. Moreover, relative to some reactive species (ROS, reactive oxygen species: RNS, reactive nitrogen species), the metabolites are more effective scavengers than melatonin itself. ^1^O_2_ superoxide anion radical; H_2_O_2_, hydrogen peroxide; ^•^OH, hydroxyl radical; NO^•^, nitric oxide; ONOO^−^, peroxynitrite anion; ^1^O_2_, singlet oxygen; LOO^•^, lipid peroxyl radical. MnSOD, manganese superoxide dismutase; CuSOD, copper superoxide dismutase; GPx, glutathione peroxidase; GR, glutathione reductase; CAT, catalase; γ-GC, gamma-glutamylcysteine synthase.

Melatonin also indirectly reduces oxidative stress by upregulating antioxidant enzymes [65], downregulating pro-oxidant enzymes [66], and stimulating the synthesis of other endogenous antioxidants, e.g., glutathione [67]. While the direct free radical scavenging actions of melatonin and its metabolites are generally considered to be receptor-independent, its actions on the redox-related enzymes require the intervention of receptors (Figure 1). The deacetylation and activation of mitochondrial superoxide dismutase (SOD2) also requires the stimulation of sirtuin 3 (SIRT3), a major multifunctional NAD+-dependent histone deacetylating enzyme located in mitochondrial matrix [68]. Upregulation of SIRT3 in mitochondria has been functionally linked to delayed cellular aging including that of the oocyte [69]. Moreover, SIRT3 functions as a sensor of redox homeostasis not only in the ovarian granulosa cells but also in the pre- and post-ovulated oocyte and in embryos early in their development; on the basis of these findings, it is hypothesized that SIRT3 plays an active role in determining female fertility due to its protection of oocytes as they mature and age [70,71].

**Figure 2:**
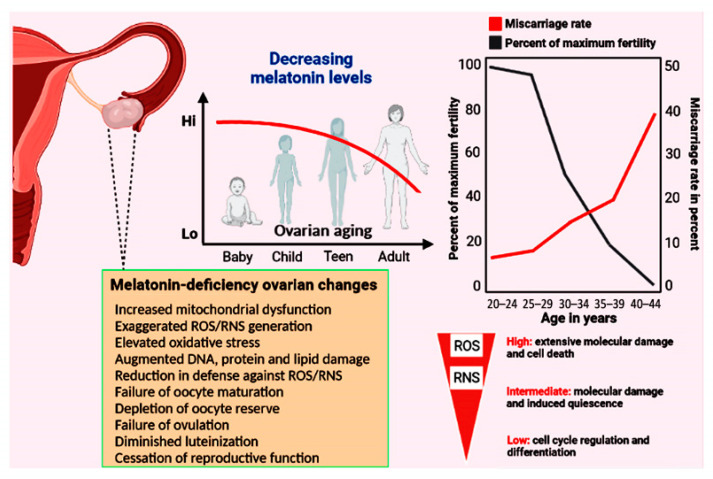
The top panels show the relationship between decreasing melatonin levels and the percent reduction in maximal fertility and the incidence of miscarriage as a function of age. At the time of menopause, total body melatonin levels have fallen to approximately half those in young, reproductively competent women. Among other functions, melatonin acts as a powerful direct radical scavenger and also indirectly reduces oxidative destruction by stimulating many antioxidative enzymes (see Figure 1). Considering the multiple protective actions of melatonin in limiting the accumulation of oxidatively damaged molecules during aging generally, it has often advanced as an anti-aging molecule. In the current report, we propose that the accumulated damage to key ovarian components due to the loss of this high-protective molecule contributes to infertility and reproductive cessation. The lower left panel summarizes some of the ovarian changes that have been reported when melatonin is not available in adequate amounts. Low levels of free radicals actually function as signalling molecules, but elevated levels mutilate DNA, proteins, lipids, etc. The majority of free radicals are produced in mitochondria; current evidence indicates that melatonin is synthesized in the mitochondria of ovarian cells so it is perfectly positioned to scavenge the continually produced reactants thereby providing protection against cellular dysfunction and infertility.

Because of its location and likely synthesis in the mitochondria of perhaps all cells [33,34,72,73], melatonin is ideally situated to provide protection against oxidative stress since these organelles are a major site of free radical generation [74] (Figure 2). Many immunochemical investigations have confirmed melatonin’s ability to incapacitate free radicals in the mitochondria [75] and it does so more effectively than the well-known antioxidants, vitamins C or E [76]. Even compared to the chemically modified vitamin E (Mito E), which accumulates in high concentrations in mitochondria, melatonin is equal to or better than the synthetic product in protecting cells from damage due to exposure to lipopolysaccharide plus peptidoglycan, two highly damaging bacteria-derived, mitochondrial toxins [77]. Because of its uncommonly high efficacy in shielding molecules from free radical-mediated disfigurement, it has been designated a mitochondria-targeted antioxidant [78,79,80].

As noted above, many of the metabolites that are produced when melatonin neutralizes a free radical are similarly efficient in doing so [73,81,82,83] (Figure 1). Thus, there is a long chain of reactions that allows melatonin and its products to function in the control of redox homeostasis at the level of the mitochondria, in lipid-rich membranes and throughout the cell [33,84,85]. Moreover, cells have enzymes that repair damaged DNA with these regenerative processes being enhanced by melatonin [86]. As summarized by Galano and colleagues [85], melatonin and its metabolites work via multiple means to neutralize free radicals and protect against oxidative stress and cellular/mitochondrial dysfunction; these actions include radical adduct formation, hydrogen atom transfer, electron transfer, etc. Beyond this, melatonin and its metabolites activate enzymes that convert highly damaging radicals to less destructive products and reduce the formation of ROS/RNS by inhibiting pro-oxidant enzymes (Figure 1).

Melatonin’s protection of membranes from lipid breakdown has critical benefits for maintenance of normal cell physiology by determining membrane viscosity and influencing the development of lipid rafts [87,88,89]. Lipid rafts are present in all eukaryotic cell membranes and consist of transient lipid and protein complexes that float freely in membranes and coalesce to form microdomains; these specialized areas are critical in terms of sorting and signaling processes and help to maintain cells in an optimally functional state [90]. Oolemmal microdomains which include ganglioside-enriched lipid rafts provide a site for sperm docking and fertilization in the post-ovulatory oocyte [91]. Lipid rafts are also essential in underpinning phase separation of biomolecular condensates, of which there a variety of types in the cytosol, nucleoplasm and mitochondrial matrix [92]; these transient condensates are of functional importance in the oocyte physiology [93]. For a comprehensive evaluation of the role of bimolecular condensates on cell physiology and how they may be impacted by free radicals and influence reproductive physiology, the reader is urged to consult the recent reviews related to this subject [92,94].

It has recently been proposed that females are equipped to produce more melatonin than males because they have a slightly higher reserve/maximum capacity related to the gender bias expression of both acetylserotonin methyltransferase (ASMT) and ASMT-like (ASMTL) genes [95]; ASMT is the final enzyme in the synthetic pathway for melatonin. The mitochondria of all cells are believed to produce extrapineal melatonin [33,34], which under normal circumstances is not released into the circulation as is that produced by the pineal gland [96]. Considering the proposed role of melatonin in maintaining longevity and delaying disease onset in the aged [97], along with the generally greater lifespan of females than males [98], it was proposed that females survive to a more advanced age because they have a higher functional reserve for melatonin synthesis [95]. The proposed dimorphism with the supposed higher melatonin levels in females than males does not, however, prolong the reproductive span of females over that of males since females lose the capacity to reproduce much earlier in life when compared to males which maintain functional gametes to a more advanced age. There are multiple processes, however, that determine the rate of biological aging, cessation of reproduction, and the progression of AD which are likely independent of the melatonin status.

The greater potential for higher melatonin levels in females could come into play when highly stressful, ROS-producing reproductive processes occur. For example, high levels of melatonin would be advantageous at the moment of ovulation given the release of ova is associated with elevated free radical levels [99,100]. Likewise, nighttime melatonin levels in rats progressively increase as parturition approaches with these values falling to normal levels immediately after the birth of the young [52]. The elevated melatonin at the time of parturition, which is a major stressor, may be designed to protect against oxidative damage to the mother and the fetus. Alternatively, the higher melatonin levels in late pregnancy may be related to melatonin-mediated synergism with oxytocin to induce stronger uterine contractions required for delivery [38]; this would be consistent with the time of human parturition which most often takes place at night [101], when circulating melatonin levels are routinely elevated.

## 4. Physiological/Biological Ovarian Aging: Protection Using Melatonin

Every organ shows signs of aging and functions less efficiently in advanced age. The female reproductive tract is one of the first systems to exhibit hallmarks of aging. This deterioration has become increasingly significant since women in industrialized societies are now more often trying to become pregnant near the end of their reproductive life span (>35 years of age) [102]; this increases the possibility of failure to conceive, leading to experiences of miscarriage or giving birth to offspring with a physical or genetic disability. Age-related changes in the female peripheral reproductive system are similar to those that occur in reference to general systemic aging [103]. The rate of aging, however, exhibits some plasticity with different individuals of the same species or organs deteriorating at different rates. In addition, species vary extraordinarily widely in reference to the maximal average duration of survival and, therefore, also in their reproductive lifespan [104].

While males renew spermatogonial cells throughout the majority of their life, when females are born, they are endowed with a finite number of oocytes (an estimated 400,000) which is not amplified during their lifetime [105] although there is a modicum amount of data that suggests otherwise [106]. This oocyte pool is represented by the total number of primordial follicles present in the ovarian cortices [107]. Due to the continual pre-established process of atresia of existing oocytes, the number of primordial follicles falls to an estimated 1000 at menopause [108]. The rate of oocyte attrition from birth to menopause is continuous and is not associated with an abrupt change as menopause approaches. The few oocytes that do not undergo atresia and/or apoptosis are recruited for active growth with their eventual ovulation. The molecular mechanisms for follicle growth and oocyte maturation involve the signaling molecules mammalian target of rapamycin (mTOR) and AKT (protein kinase B) as the initiators of these processes [109]. During the reproductive life span, in broad terms, fertility generally correlates with the residual quantity of oocytes remaining in the ovaries (the ovarian reserve) [110] although a successful pregnancy also depends on the availability of high-quality oocytes [111]. Typically, the loss of fertility for women becomes apparent during the fourth decade of life (Figure 2).

As with many other degenerative processes, ovarian aging is determined to a significant degree by the levels of oxidatively damaged molecules that are produced in ovarian cells [112]. The aging phenotype, however, is far more complex than being caused by a single process and occurs because of a gradual loss and subsequent extinction of what is considered normal ovarian physiology. In general, ovarian aging is categorized into one of two types, i.e., either physiological or pathological [113]. The former of these merely reflects the gradual deterioration of multiple cellular changes that occur in the ovary which ultimately cause the failure of ovulation and menopause onset. In contrast, reproductive aging can also be pathological, where ovarian function ceases earlier than normal and is associated with diminished ovarian reserve (DOR), premature ovarian insufficiency (POI), or, in the case of in vitro fertilization–embryo transfer (IVF-ET), a poor ovarian response (POR) to hormone treatment. There are a wide variety of pathogenic factors that contribute to pathological ovarian aging, many of which are man-made [114], but all of which include oxidative stress.

Under physiological conditions, there is a delicate equilibrium between generation of oxidatively damaging molecules and the ability of the defense mechanisms to negate the offending processes. Clearly, however, these two processes are not always balanced since some oxidative damage always occurs which causes cellular and organ deterioration, i.e., aging. ROS play a major role in tissue degradation, including during ovarian aging; to a lesser extent, RNS are also involved. Collectively, ROS and RNS are frequently referred to as free radicals, although structurally not all of them are in fact free radicals.

The loss of cells due to apoptosis is a major feature of ovarian aging [115]. Both the exogenous and endogenous apoptotic pathways can be activated and normally occur during physiological aging of the ovary [116] (Figure 1). Oocyte apoptosis obviously diminishes the total number of gametes available for ovulation. When granulosa cells are lost, significant changes occur in the nutrient supply to the oocyte and other cells and is associated with alterations in the ovarian cellular microenvironment [117].

Chronic low-grade inflammation is often associated with aging including that of the ovary [118]. Inflammation occurs simultaneously with oxidative damage since ROS stimulate the NLRP3 inflammasome, thereby recruiting inflammatory cells which in turn produce pro-inflammatory cytokines., e.g., IL-1β and IL-18. In addition, NF-ĸB is activated by ROS that enhance inflammation which further upregulates the NLRP3 inflammasome. These processes culminate in immune system activation which aggravates oxidative damage.

**Figure 3:**
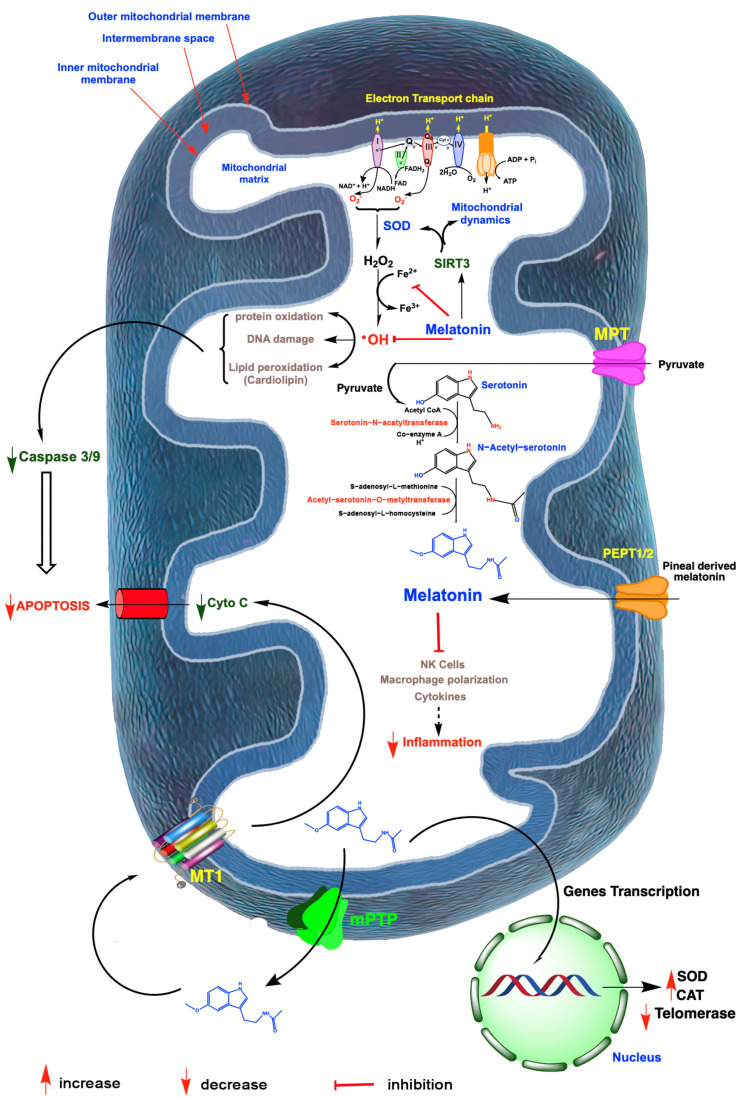
A summary of free radical generation in mitochondria and the role of melatonin in mitigating oxidative damage and ovarian aging. Radicals are generated especially as a result of electron leakage from the electron transport chain in the inner mitochondrial membrane; the rogue electrons chemically reduce adjacent oxygen molecules to produce the superoxide anion radical (O_2_^•−^). This reactant is quickly dismutated by superoxide dismutase 2 (SOD2) to hydrogen peroxide (H_2_O_2_) or it couples with nitric oxide to produce the highly oxidizing peroxynitrite anion (ONOO^−^; not shown). H_2_O_2_ is converted to the hydroxyl (^•^OH) radical via the Haber–Weiss reaction, which is kinetically slow, or via the Fenton reaction, both of which require a transition metal such as ferrous iron (Fe^2+^). The ^•^OH, along with other oxidants, damage molecules, which initiate apoptosis. The antioxidant, melatonin, which is synthesized by a number of ovarian cells, likely in the mitochondria, as well as pineal-derived melatonin which enters these organelles, chelates iron and other redox reactive transition metals. Via the activation of sirtuin 3 (SIRT3), melatonin also upregulates SOD2 and impacts mitochondrial dynamics in favor of renewing mitochondria. Finally, melatonin directly neutralizes ^•^OH and the ONOO^−^. Via these combined actions, melatonin serves as a powerful protector of mitochondrial integrity and preserves optimal cellular function which delays ovarian aging. Melatonin also functions as an anti-inflammatory which, especially when chronic, compromises mitochondrial physiology leading to ovarian cell, including oocyte deterioration. Mitochondria produced melatonin also escapes these organelles to act on melatonin receptors (MT1) in the mitochondrial membrane, which reduces the release of cytochrome C (Cyto C) thereby inhibiting programmed cell death which would otherwise advance ovarian aging. Finally, in the event of ovarian cancer, melatonin impedes the synthesis of telomeres by reducing telomerase activity thus slowing cancer cell renewal. I-IV; mitochondrial complex.

Mitochondria are involved in essentially every ovarian cell process and are the source of many of the free radicals that a cell generates. Mitochondria also have their own DNA (mtDNA) and, in contrast to nuclear DNA, it is in jeopardy of being readily damaged because it lacks histone protection and, of equal or greater importance, it is in the immediate vicinity of the numerous ROS that are formed during oxidative phosphorylation (OXPHOS) [119,120] (Figure 3). The problem is further compounded by the fact the ROS-mediated mtDNA damage disrupts ETC processes and also promotes abnormal mtDNA-protein crosslinking, both of which further accelerate the deterioration of optimal mitochondrial physiology due to an increasing number of oxidatively damaged molecules [121]. Thus, the functional damage of mitochondria becomes a vicious cycle of degradation.

Information related to the molecular events that induce the obvious alterations in diminished ovulation and advanced menopause onset still lack many details [122,123]. These changes do, however, coincide with the reduction in circulating nocturnal melatonin levels as well as the presumed loss of melatonin production in the mitochondria of the ovarian cells [72,124] (Figure 2). This is relevant since the mitochondria play a central role in oocyte aging and the reduction in melatonin in this organelle significantly influences their function; the mitochondria of pathological cells have melatonin concentrations approximately only half those of healthy cells [125,126]. Such greatly depressed levels of this important mitochondrial reducing agent likely allow for the more rapid accumulation of oxidatively damaged molecules. Mitochondrial dysfunction in the aged oocyte contributes to failure of oocyte development, successful fertilization, satisfactory uterine implantation and embryonic development. Given that mitochondrial function determines the bioenergetic status of the oocyte, it is obvious that OXPHOS and ATP production in these organelles must be optimal to prevent arrested oocyte growth and the misalignment of chromosomes [127]. During aging, the oocyte mitochondrial membrane potential exhibits a gradual decline which negatively impacts energy production [128] (Figure 3). The respiratory capacity of mitochondria obtained from oocytes recovered from older ovaries is depressed, which leads to reduced ATP production; ATP is the energy currency required for all aspects of a healthy ovum and the zygote [129,130,131].

The functional state of the mitochondria also determines the overall aging rate and quality of the oocyte which impacts fertility, mitochondrial fusion, which is regulated by two major mitofusins (MFN1/2) and dynamin-like 120 kDa protein (OPA1), and prevails aiding in the maintenance of healthy mitochondria. On the contrary, when mitochondria undergo fission, they exhibit membrane depolarization, depressed ATP production and are usually destroyed by mitophagy [132]. Like other molecular processes, mitophagy also diminishes with age, thereby preserving more dysfunctional mitochondria and contributing to fewer healthy oocytes [133]; less than optimally functioning oocytes are incompatible with successful reproduction.

Changes in the nuclei are also recognizable during ovarian aging. Telomeres, i.e., tandem DNA repeat sequences, are extensions of all linear chromosomes of eukaryotic cells. In most cells, telomeres become shorter with each cell division. Oocytes are endowed with the enzyme telomerase which, to a degree, may function in maintaining telomere length [134]. The length of the telomere is closely correlated with the duration of time in which females remain capable of reproducing as well as their life span [135]. Telomerase is a reverse transcriptase that maintains the correct DNA replication of the telomeres. The length of the telomere of somatic cells seems to be useful to predict the stage of reproductive aging with longer telomeres correlating with late-onset menopause [136]. Specifically, telomere length in human oocytes is an indication of the quality of the ovum with the general conclusion that telomeres play a significant role in maintaining genomic stability since diminished telomere length is associated with the number of chromosomes in embryonic cells being less or more than the haploid number, i.e., aneuploidy [137]. In addition, women with premature ovarian insufficiency (POI) have reduced levels of telomerase in the ovarian granulosa cells [138]. Thus, depressed telomerase activity or shorter than normal telomeres in select ovarian cells contribute to poor quality oocytes that compromise reproductive capacity.

Although the outcomes of the published reports have not always been consistent, locally produced or pineal-derived melatonin may have an influence on steroidogenesis in the human ovary. This contrasts with observations on photosensitive, seasonally breeding animals where melatonin has a profound effect on all aspects of ovarian function [139]. In non-seasonal breeders such as the human, however, the actions of melatonin indirectly or directly on ovarian physiology as it relates to aging are minor [140]. Four decades ago, several studies documented that melatonin stimulates the in vitro secretion of progesterone and 17β-estradiol by the mammalian ovary, including in the human [141,142]. The impact of melatonin on progesterone production has been recently confirmed. The most thorough investigation related to melatonin and ovarian steroidogenesis, particularly progesterone, originates from the work of Fang and colleagues [143]. This group reported that melatonin upregulated steroidogenic acute regulatory protein (StAR) in primary cultures of human granulosa/lutein cells collected from women undergoing in vitro fertilization. Progesterone is synthesized in mitochondria from cholesterol with the entrance of cholesterol into the mitochondrial matrix being mediated by StAR; thus, StAR is rate-limiting in progesterone production. Fang et al. [143] determined that melatonin upregulated StAR with this response being mediated by its membrane receptors (on the cell membrane or on the mitochondrial membrane?) and involved activation of the PI3K/AKT pathway. As a result of melatonin stimulation, progesterone levels in the incubation medium more than doubled and during the IVF procedure, melatonin levels in the follicular fluid were positively related to serum progesterone concentrations.

Elevated oxidative stress in the ovary also negatively impacts progesterone production as shown by Taketani et al. [144]. As observed by Fang et al. [143], this group reported that melatonin concentrations in human ovarian follicular fluid were positively correlated with progesterone levels and negatively correlated with 8-hydroxy-2′-deoxyguanosine (8-OHdG), a reliable marker of oxidatively damaged DNA. When luteinized granulosa cells obtained from women during IVF were incubated with the oxidant, H_2_O_2_, progesterone production was diminished. The addition of the free radical scavenger and indirect antioxidant, melatonin, in the incubation medium overcame the suppressive effect of H_2_O_2_ on progesterone levels and also reduced 8-OHdG concentrations. Judging from the published reports, melatonin may have a distinct role in influencing ovarian progesterone synthesis. It should be noted, however, that granulosa cells exist in many stages of differentiation. The current human studies cited herein were conducted on mature granulosa cells. Whether melatonin would have the same actions on less differentiated granulosa cells has yet to be tested. Despite the data summarized herein, there is not uniform agreement regarding the regulation of progesterone production by melatonin [143].

The granulosa cells are also the major site of ovarian estrogen synthesis and secretion [145]. In an extensive retrospective study of infertile women, Yuzko et al. [146] reported that melatonin treatment caused a significant rise in circulating luteinizing hormone levels without a commensurate increase in blood estrogen levels. Melatonin treatment also was associated with depressed levels of anti-mullerian hormone; this latter finding indicated that the infertile woman did have a diminished oocyte reserve as expected, with this marker not being influenced by melatonin. The significance of these findings is difficult to evaluate since the causes of infertility in these patients varied widely. In essence, there is a paucity of information related to any role locally produced estrogen may have on ovarian aging.

Via its actions on ovarian steroid production, melatonin may impact the time of menopause and delay the climacteric. Thus, by involving steroidogenesis, melatonin could indirectly impact reproductive cessation in human females. Nevertheless, a direct action of progestens or estrogens on ovarian aging *per se* has little experimental or clinical support.

As a consequence of increasing age, the ovaries exhibit a gradual loss of the total oocyte number and a reduction in their quality. The result is a reduced oocyte reserve and/or premature ovarian insufficiency [147]. Senescent changes in the female reproductive system are also associated with an increased likelihood of congenital birth defects in the offspring, for example, the appearance of spina bifida or trisomy 21 in the newborns [148]. The former of these conditions is a result of several factors including nutritional, genetic and environmental factors and a vitamin B9 (folate) deficiency [149], while trisomy 21 (Down syndrome) is a consequence of non-disjunction during oocyte meiosis such that all cells in the offspring end up with three copies of chromosome 21 [150].

## 5. Melatonin: Regulation of Redox Homeostasis for Delaying Ovarian Aging

Melatonin’s protective effects on the ovary, as in other organs, is mediated by both receptor-mediated processes as well as receptor-independent means [36] (Figure 1). The latter occurs when melatonin and its metabolites function as direct free radical scavengers while the former involves receptor-mediated stimulation of antioxidative enzymes or inhibition of pro-oxidative enzymes. It is likely that both pineal-derived melatonin, which is extracted from the blood, and well as locally produced melatonin by granulosa cells and oocytes normally participate in protecting ovarian tissues from age-related deterioration. A significant portion of this protection is lost as age progresses, however, since the concentrations of melatonin in the blood and in the ovarian follicular fluid fall substantially leaving the surrounding tissues increasingly vulnerable to oxidative damage which contributes to the cessation of reproductive capability [52,151]. In addition to the depletion of available melatonin to protect against oxidative stress, the number of melatonin receptors diminishes in the aged ovary thereby limiting the upregulation of antioxidant enzymes [40]. The drop in melatonin also makes the ovary more likely to become inflamed [152]. Both persistent low-grade inflammation and accumulated oxidative damage eventually interfere with oocyte maturation and ovulation leading to the onset of menopause and the termination of fertility. Elevated oxidative stress and prolonged inflammation contribute to an increased frequency of genetic mutations which accelerate ovarian aging [153]. Concurrently, the hormonal patterns that govern ovarian cyclicity and successful ovulation are also altered [154].

A variety of events conspire to reduce oocyte availability and maturation as menopause approaches. The age-associated reduction in healthy oocytes is in part a consequence of atresia, a process that is common but not well understood [155]. A second cause of the persistent loss of available oocytes is apoptosis; these two processes eventually lead to a diminished ovarian reserve (DOR). Women with DOR may still have a menstrual cycle but they are typically infertile. By the time of menopause, the primordial follicle pool containing immature oocytes is reduced to several hundred from the many thousands that were present at menarche. This reduced number of follicles signals an elevation in the rate of loss of the few remaining follicles; as a result, at the end of the menopause period, follicle number is nearly totally depleted [156].

Whereas the molecular processes related to menopause are well studied, the events that cause ovarian aging have been less thoroughly investigated. They have, however, many common features. Since it is not uncommon in today’s societies for a woman to delay having a child until late in her reproductive life and because of the number of serious diseases that often occur during the post-menopause period, there is intense interest in procedures/treatments that delay ovarian aging and prolong female reproductive life [157].

Proposed pathophysiological processes that have been implicated in ovarian aging are multiple, such as the accumulation of disfigured molecules resulting from oxidative stress including damaged DNA, increased number of mutations, molecular errors that accompany meiosis, telomere shortening, mitochondrial malfunction which reduces ATP generation while concurrently increasing ROS formation, and disordered cytosolic protein metabolism [103,158,159]. Identifying ways to delay or reduce the degenerative changes in the ovarian reserve of women nearing menopause may safely extend their reproductive life span, prevent the likelihood of giving birth to offspring with genetic deficits, improve the success rate of women who undergo in vitro fertilization–embryo transfer (IVF-ET), and delay POI, also known as premature ovarian failure (POF) or premature menopause. POI is identified in woman under the age of 40 years and is likewise associated with an earlier than normal reduction in oocyte number as well as early menopause [160]. While DOR and POI are described as distinctly different conditions, there is significant overlap in their clinical presentations and in the underlying causes [161], so any potential treatments could have benefits for both conditions.

Considering the major role that oxidative stress plays in the cessation of successful gametogenesis and in ovarian decline [113], the administration of a multifunctional, mitochondria-targeted antioxidant in an attempt to maintain the ovarian reserve and preserve healthy oocytes may be a good choice [78,79,80]. Melatonin meets these specifications. Because of their high energy requirements, oocytes are especially enriched with mitochondria where their number is greater than in any other mammalian cell [162]. The shunting of electrons between the complexes of the electron transport chain (ETC), which is required for eventual ATP synthesis, also leads to the formation of an abundance of ROS when electrons are leaked from the ETC and reduce neighboring oxygen molecules to the superoxide anion radical (O_2_^•−^) [163] (Figure 3). Considering the high reactivity of free radicals and, in many cases, their very short half-life, it is imperative that oocyte mitochondria are equipped with adequate endogenous antioxidative defense mechanisms to prevent damage to this critically important cell. Melatonin, a highly efficient inhibitor of oxidative stress, is synthesized in the oocyte [46], particularly in the mitochondria [45]. As a result, melatonin is in the optimal position to prevent oxidative mutilation of the oocyte. Additionally, pineal-derived melatonin circulating in the blood gains access to cells via several routes [164,165] (Figure 3). It is likely, however, that the continually synthesized and potentially inducible mitochondrial melatonin has a much greater role in protecting the oocyte from ROS-mediated damage considering that melatonin in the blood is only available during darkness when it is being produced by the pineal gland [166]. Unfortunately, aging is associated with a gradual loss of melatonin in the blood and its likely diminished production in the oocytes (and in granulosa cells) leaving the follicles in a highly vulnerable situation at a time when ETC efficiency is waning and free radical generation is increasing [167]. We speculate that the reduction in melatonin and the simultaneously elevated ROS generation in the ovary accounts, in part, for reproductive failure and menopause onset (Figure 2).

When ovarian aging is considered, it is usual that the viable primordial follicle stockpile and the number of oocytes that are biologically capable of maturing to undergo ovulation, fertilization and embryo development are the major concern [159,168]. Very closely physiologically related to these processes is the functional state of the granulosa cells [169]. During the reproductively capable period, the granulosa cells undergo morphophysiological changes typical of functional decay accompanied by a reduction in superoxide dismutase 1 and 2 and in catalase making them less capable of resisting free radical damage [170]. This increases the frequency of mitochondrial DNA mutations and is accompanied by the upregulation of glutathione S-transferase theta 1. This enzyme also exaggerates oxidative stress since it catalyzes the conjugation of chemically-reduced glutathione to a variety of compounds; glutathione is an important intracellular antioxidant that is typically in high concentrations in granulosa cells [171]. As with the oocyte itself, the granulosa cells also synthesize the antioxidant melatonin, but as these cells age, they lose that capability just as other tissues do. Thus, not only does the oocyte decay, but the supporting cells do as well, which leads to a lower reproductive capability.

The ovarian stroma surrounds each follicle during its development and plays an essential role in the maintenance of a healthy follicle/oocyte unit [172]. During its maturation, a follicle moves from the collagen-rich cortex to the less dense medulla and eventually back to the cortex for ovulation. The mechanics of this movement and the cellular composition of the stroma during these movements significantly determines the functional state of the follicle and its oocyte [173]. This was proven by in vitro studies where follicles were grown in different stromal compositions which generated different extracellular matrices (ECM) that differentially impacted the rate of follicle maturation [174]. During aging, the ECM of the stroma changes substantially relative to its viscoelastic properties [175]. In general, the stroma generally becomes more rigid as menopause approaches, which may restrict the movement of follicles to the surface for ovulation. Among a number of other alterations in the stroma, there is also a change in the ratio of M1/M2 macrophages in favor of the M1 proinflammatory cell type contributing to what is referred to as inflammaging [176], an age-associated change related to aging that is not unique to the ovarian stroma since it occurs in many other tissues as well. How precisely this low-grade inflammation alters the rate of reproductive decay remains to be determined, but, as inflammation always does, it increases the amount of oxidatively damaged stromal elements, rendering them less functional (Figure 3).

Sirtuins, a family of highly conserved histone-deacetylating enzymes that are differentially distributed in cells, are frequently described as influencing aging and longevity pathways [69]. The roles of these critically important enzymes, with the exception of SIRT1, have not been extensively investigated relative to senescent processes of the ovary. SIRT1 knockout mice survive but are severely compromised relative to their ability to reproduce. SIRT1 also influences oocyte maturation and quality. For example, pharmacological inhibition of SIRT1 interferes with meiosis and those that continue on to meiosis II are defective in terms of spindle formation and chromosome misalignment [177]. While SIRT2 has been rather extensively investigated in relation to somatic cell division, there is a dearth of information on its actions in gametes [178].

Mitochondrial SIRT3 protein has been identified in human in all components of the developing follicle, but knockout of this deacetylase in mice does not interfere with successful reproduction. Despite the absence of SIRT3, these gametes can be successfully fertilized, at least in vitro. This is somewhat unexpected since oocytes with a deficiency of SIRT3 exhibit elevated levels of oxidative stress [179]. SIRT4 is a weak deacetylase; as a lipoamidase [180], it reduces the activity of the mitochondrial enzyme pyruvate dehydrogenase complex which limits mitochondrial acetyl-coenzyme production. This, in turn, may lower mitochondrial melatonin synthesis [72]. Overexpressed SIRT4 in oocytes causes spindle and chromosomal disorders and altered mitochondrial physiology with elevated ROS generation [181]. How or whether these changes relate to oocyte physiology, however, is undefined. SIRT6 curtails oocyte DNA damage mediated by oxidative stress and also functions in stabilizing their telomeres. With a deficiency of this deacetylase, aged oocytes have shorter telomeres, an indication of aging-related decay [182]. SIRT6 is also involved in maintaining redox homeostasis via its influence on Nrf2 [183]. Even less is known about the functional significance of SIRT5 or SIRT7 to peripheral reproductive physiology. In oocytes, as in somatic cells, advancing age is associated with diminished mitochondrial function which causes increased oxidative stress [184]; this also contributes to potential deficiency in the NAD+-dependent deacetylase activities, thereby preventing healthy oocyte aging (Figure 2).

An interaction between melatonin and SIRT3 has clear implications for ovarian function as uncovered in several recent studies. In humans, obesity is a negative factor for the successful fertilization of the oocyte by the sperm with the mechanisms for this inhibition probably being multifactorial but only ill-defined [185,186]; excessive body weight is believed to contribute to the decline in female fertility. NAD+ (nicotinamide adenine dinucleotide) is a necessary co-factor for SIRT3 and for its regulation of mitochondrial redox homeostasis [187] (Figure 3). Feeding female mice a high-fat diet that promotes obesity reduces their fertility, which is accompanied by a significant drop in NAD+ levels in whole ovaries and meiosis II oocytes [188]; as anticipated, MitoSOX immunocytochemistry documented the elevated levels of ROS in the oocyte mitochondria, along with a reduced membrane polarization and a depression of genes related to oxidative phosphorylation. Supplementing the obese mice with nicotinamide riboside, a precursor of NAD+, elevated the transcription of SIRT3 and protected the oocyte from oxidative stress.

Using the same obese mouse model, we had previously reported that feeding a high-fat diet caused the acetylation of SIRT3 in oocytes with extensive oxidative damage to these organelles [189]. Conversely, treating these animals orally with melatonin caused the deacetylation of SIRT3, which allowed for the deacetylation and activation of SOD2 [96], leading to a significant reduction in the exaggerated oxidative stress measured in the oocytes of the obese animals. Finally, the use of morpholino knockdown of SIRT3 expression further confirmed the essential role of the SIRT3/SOD2 pathway in maintaining viable oocytes in obese animals and re-emphasized the importance of melatonin in regulating this axis (Figure 3).

## 6. Delaying Infertility with Melatonin

Very shortly after the discovery of melatonin as a potent endogenously generated radical scavenger [58], melatonin was proposed as a potential agent for delaying systemic biological aging [190]. This suggestion was made because a primary theory of aging is that the life-long accumulation of oxidatively damaged molecules interferes with cell physiology throughout the organism, thereby contributing to biological deterioration of all organs [191,192]; also, changes in pineal physiology was suggested to relate to aging [193]. In addition to its documented direct free radical incapacitating activity of melatonin, it was soon discovered to also stimulate neural glutathione peroxidase, a major ROS detoxifying enzyme [194], which further supported the idea that melatonin may defer oxidative decline and enhance longevity.

The theory suggesting melatonin may be an important component of the system that leads to senescence was consistent with the known marked drop in melatonin levels in aged animals and humans [167,195,196,197]. Of additional interest is that the loss of melatonin during aging was prevented by restricting the daily calorie intake of rodents, a treatment well known to preserve the function of all organs including the reproductive system and to significantly prolong life [198,199]. The fact that calorie restriction significantly delays aging and simultaneously preserves pineal melatonin synthesis suggested that the function-preserving effects of calorie restriction may, at least in part, be a consequence of the conserved levels of the anti-aging molecule, melatonin [200]. Early-life pineal gland removal from rats, which eliminates the circadian melatonin rhythm thereafter, is associated with an accelerated accumulation of oxidative stress in multiple organs again indicating that the melatonin, considering its circadian rhythm properties may have a role in delaying age-related deterioration and enhance longevity [201].

Beginning approximately 15 years ago, Tamura and colleagues embarked on a series of studies to identify the potential importance of melatonin as a protective agent against ovarian aging [52]. In their initial study, mice were administered supplemental melatonin in their drinking between the age of 10 and 43 weeks. Administering melatonin via this means is considered essentially physiological since mice are nocturnal and drink the bulk of their fluid at night; therefore, the nocturnal intake of drinking fluid also causes a robust nighttime elevation in circulating melatonin as occurs normally [202]. At the end of the 43-week period, ovaries and oocytes were retrieved and examined for their developmental status. In the control mice not given supplemental melatonin, there were fewer follicles at all stages of development, i.e., primordial to antral follicles [52]. The ovaries of mice given melatonin also had more ovulations as indicated by the number of corpora lutea. Microarray examination of gene expression showed 77 ovarian genes decreased with age in the control but not in the ovaries of mice supplemented with melatonin. Furthermore, molecular pathway analysis indicated that the additional melatonin preserved ribosomal function, accurate protein synthesis and optimal gene transcription, all features consistent with conserved ovarian physiology and delayed aging of these organs. Finally, they determined that the total antioxidant capacity of the ovaries from melatonin-treated mice was maintained at a higher level along with longer telomeres and improved expression of SIRT1 and SIRT3 genes, which are often described as longevity-promoting genes [52].

In reference to other molecular measures performed, the level of damaged DNA (8-hydroxydeoxyguanosine; 8-OHdG) was lower in the ovarian extracts of mice that had consumed melatonin. The authors speculated that the reproductive efficiency was improved after the extra melatonin was taken by the ovaries where it reduced oxidative stress and improved oocyte quality allowing for successful fertilization, implantation and the maintenance of pregnancy [52]. Collectively, the comprehensive findings of Tamura and co-workers strongly support the idea that melatonin is a significant agent in deferring the rate of ovarian aging [52].

In the same report, Tamura and co-workers examined the ability of melatonin to improve the fertility of women undergoing in vitro fertilization–embryo transfer (IVF-ET) [52]. Infertile women with poor oocyte quality (maximum of 50% fertilization rate) were either treated with melatonin (3 mg daily at bedtime) or not for 30 days; immediately at the completion of the treatment period, oocytes were retrieved from all patients for IVF-ET. Even with the short duration of treatment and the low dose of melatonin, the patients who ingested melatonin had both an improved fertilization and an elevated pregnancy rate relative to the subjects not given this agent. It seems likely that the use of higher doses of melatonin and a longer treatment period would further improve the fertilization rate of otherwise infertile women.

The results summarized in several subsequent publications [203,204,205] and a clinical trial [206] are in agreement with the findings of Tamura and colleagues, related to the ability of orally ingested melatonin to improve oocyte quality and pregnancy outcomes in women [52]. The registered clinical trial was randomized and double-blinded with the treatment being a repeat of that utilized by Tamura and co-workers., i.e., a one-month treatment with 3 mg melatonin (32 women) or not (34 women) [52]. As to the outcomes of the assisted reproductive measures, melatonin-treated women had a greater number of mature MII oocytes and an improved quality of embryos (grades 1 and 2). Again, it is the feeling of the authors that the amount of melatonin utilized in this trial was probably the least effective dose with larger amounts likely yielding even greater reproductive improvements. Recently, Showell et al. [207] reported that there is limited evidence in support of supplemental oral antioxidants for subfertile women, among them melatonin. Clearly, there is an urgent need for additional studies with pharmacological doses of melatonin considering the substantial rise in female infertility throughout the world. The findings related to the improvement of reproductive outcomes after melatonin treatment are summarized in two recent reviews [208,209].

The first investigation specifically designed to determine the effect of exogenously-administered melatonin in slowing age-related ovarian degeneration was only recently performed [210]. As a rationale for these studies, the authors noted that there are a significant number of publications suggesting melatonin delays organismal aging which could also translate into a similar slowdown in the decline of the reproductive system [190,211,212]. Moreover, daily supplemental melatonin had been shown to delay age-related changes in other organs [213,214,215] and the concentration of melatonin in the ovarian fluid is greater than that in the blood, indicating it is likely of some importance for maintaining normal ovarian physiology [20,41]; in follicular fluid, melatonin functions as an antioxidant [21]. Finally, the gradually diminishing amplitude of the blood melatonin rhythm which, by the time of menopause, is significantly lower than that in younger fertile women, possibly contributes to the cessation of menstrual cyclicity and ovarian collapse.

The publication related to the ability of melatonin to modify the rate of ovarian aging especially investigated the preservation of healthy oocytes [210]. Mice, beginning at 10 weeks of age, where administered melatonin in their drinking fluid or water only for the next 33 weeks. Near the conclusion of the investigation, recently ovulated oocytes were collected from the oviducts and incubated with sperm to test their fertilizability. Not only did melatonin treatment maintain a higher number of ovulated oocytes, which were decreased in the control animals; additionally, the collected oocytes from melatonin-treated mice were fertilized at a higher rate with successful blastocyst development. A comparative histological analysis of the ovaries also revealed more primordial, primary and secondary follicles in the ovaries from melatonin supplemented mice. The length of the telomeres was greater in the ovarian cells, the mRNA expression of autophagy-related protein light chain protein 3 (LC3) was significantly depressed relative to that in control mice while mRNA expression of SIRT1 and SIRT3 was activated following melatonin ingestion; each of these measures is an indication of cells experiencing delayed aging. Microarray analysis revealed 48 genes either related to deferred aging or antioxidant protection were also upregulated proving that the safeguarding actions of melatonin against age-related decay of the ovaries are multifactorial; in particular, melatonin treatment increased the quantity and quality of oocytes that were maintained in a healthy state beyond what occurred in non-melatonin-treated mice probably due to its antioxidant action. Other protective effects may well have been mediated by the membrane receptors resident on ovarian cell membranes. While these findings add to the body of knowledge related to mechanisms of the way ion which melatonin defers ovarian aging, and presumably systemic aging, they probably do not exhaust all the potential mechanisms by which melatonin may impede the rate of ovarian aging. As shown in studies of other organs, the processes that govern aging are diverse and exceptionally convoluted.

The most conspicuous feature of age-related ovarian collapse is infertility which is often a result of the depletion of the ovarian follicular reserve. The size of the reserve of primordial follicles varies widely among individuals with the number being strongly correlated with the number of follicles that reach the antral stage (the antral follicle count), which is required for ovulation [216]. The process of folliculogenesis beginning with the primordial follicle reserves and culminating in mature antral follicles is tightly controlled so as not to deplete available follicles prematurely. Major molecular events regulating follicle and oocyte maturation have been proposed to involve the BMP/AMH-SMAD (bone morphogenic hormone/anti-Mullerian hormone) and PI3K-AKT (tensin homolog (PTEN)/phosphatidylinositol-3 kinase (PI3K)/3-phosphoinositide-dependent protein kinase-1) pathways [217]. Perturbations of these pathways modulate (accelerate or slow down) the loss of the ovarian follicular depot; when folliculogenesis is accelerated, it exhausts the follicule/oocyte reserve which leads to premature ovarian insufficiency (POI), thereby reducing reproductive longevity with the failure of ovulation being recognized as aging.

The means by which melatonin determines the transition of primordial to antral follicles was thoroughly investigated using ovaries collected from fetal mice [218]. This group confirmed the involvement of the PI3K/AKT/FOXO3 pathway in determining the depletion of the functional follicle reserve. Unlike in the testes where germline stem cells can restore spermatogonia, in females, the follicle/oocyte reserve is essentially controlled by the rate of which these complexes mature; when growth and maturation is accelerated, it leads to the premature depletion of the oocyte depot. Yang and colleagues determined that melatonin significantly reduced the activity of the PI3K/AKT pathway which inhibited the activation of primordial follicles and their advancement to the antral state [188]. This preserved the integrity of the ovarian reserve and delayed ovarian aging; the actions of melatonin seem not to require the conventional transmembrane melatonin receptors but may have involved intracellular binding sites, e.g., quinone reductase-2, calmodulin or RZR/RORα nuclear binding, etc. [219,220].

Of additional interest is that the authors [218] showed that the granulosa cells have the enzymatic machinery required to produce melatonin and when incubated with serotonin, a necessary precursor, granulosa cells in fact synthesize melatonin. In the case of the fetal ovaries this is important, since the pineal gland of fetal rodents does not synthesize melatonin, although maternal melatonin, which readily crosses the placenta [221,222], could have mediated the inhibition of the PI3K/AKT pathway in the fetal ovaries. In an in vivo study on adult mice, the authors proved that SNAT (the rate-limiting enzyme in melatonin synthesis) knockout caused an increase in follicle activation which would normally deplete the functional oocyte reserve and led to premature ovarian aging. These findings are consistent with those of Tamura and colleagues who determined that melatonin administration delayed physiological ovarian aging possibly by modulating the PI3K/AKT pathway among other mechanisms [210].

## 7. Conclusions

The data accumulated to date suggest that melatonin may be a molecule capable of delaying normal ovarian aging and ensuring successful reproduction in humans to a later age than usual. Significant advances have been achieved in defining the molecular and cellular events associated with reproductive decline in recent years as well as the potential role of melatonin in deferring the aging of the ovary and also of the oocyte quality. For many organs and for organisms as a whole, melatonin has been frequently advanced as an age-delaying agent [223,224,225]. As animals including humans age, melatonin production typically declines markedly, which is believed to contribute to the general aging phenotype [113,226]. Given that melatonin levels gradually wane throughout life, reproductive cessation in females coincides with a time when melatonin perhaps drops to a level that is no longer compatible with ovulation and a high oocyte quality (Figure 2). While other cells can tolerate some accumulated oxidative damage throughout life, in the case of the oocyte, molecular damage is more serious since it would be disastrous in terms of successful implantation and the delivery of normal offspring [227]. This seems to be the case in late reproductive life pregnancies in humans where the frequency of functionally compromised offspring increases as menopause approaches [149] with these abnormalities being prevented by melatonin administration in experimental animals [228].

A major feature of melatonin that likely contributes to its ability to reduce molecular damage to tissues is its multifaceted function in reference to maintaining redox homeostasis [83,229,230]. Melatonin and its metabolites are all highly efficient scavengers of ROS [87,231,232]. This is particularly important at the mitochondrial level, since partially reduced oxygen derivatives are abundantly produced in these organelles and provide the basis for massive free radical damage that occurs when electrons leak from the electron transport chain [233]. To compensate for the large number of oxidizing agents produced in these organelles, mitochondria, as recently discovered, also generate their own melatonin [33,45] to aid in combatting the massive molecular destruction that would otherwise occur. Moreover, recent evidence suggests that mitochondrial melatonin production is upregulated under conditions of elevated free radical generation [234]. It is presumed that local melatonin synthesis in mitochondria of ovarian cells also diminishes simultaneous with the drop in pineal-derived blood melatonin concentrations which would mean by middle-aged, these values may only be half of what they are in younger individuals at their peak reproductive capability.

## Data Availability

Not applicable.
